# Case Report: Giant Multiloculated Pseudocystic Jejunal Leiomyosarcoma in a Dog: Atypical Morphologic Features of Canine Intestinal Leiomyosarcoma

**DOI:** 10.3389/fvets.2022.791133

**Published:** 2022-02-08

**Authors:** Mu-Young Kim, Jung Keun Lee, Kristy A. Mietelka, Hyun-Jung Han

**Affiliations:** ^1^Department of Veterinary Surgery, College of Veterinary Medicine, Konkuk University, Seoul, South Korea; ^2^Idexx Laboratories, Westbrook, ME, United States; ^3^Department of Veterinary Emergency and Critical Care Medcine, Konkuk Veterinary Medical Teaching Hospital, Konkuk University, Seoul, South Korea

**Keywords:** dog, giant, jejunal, leiomyosarcoma, necrotic cavitation

## Abstract

A 10-year-old intact female Rottweiler dog weighing 29 kg presented with 2 days history of vomiting, anorexia, and lethargy to KonKuk University Teaching Hospital, Seoul, South Korea. Ultrasonography and computed tomography (CT) scannings revealed a well-demarcated, large mass (29 × 19 × 11 cm) with numerous fluid-filled cavities. Metastases to adjacent lymph nodes were also identified on CT. This large mass and the affected intestinal segments were excised for palliative purposes. Postoperatively, the dog recovered uneventfully without any complications. The cut surface of the mass showed an exophytic growth pattern of multiloculated cystic lesions filled with serosanguineous fluid, large cavities filled with necrotic exudate, and fistulous connections between the intestinal lumen and the necrotic cavity in the mass. On histopathology, the mass was a spindle cell neoplasm expanding from the jejunal muscular layer and with pseudocystic changes. Additional immunohistochemical analysis using antibodies against smooth muscle actin, desmin, and CD-117 demonstrated that the mass was consistent with a leiomyosarcoma. Six months post-operatively, plain radiography revealed an abdominal mass, suspected to be recurrence from jejunal leiomyosarcoma. The owner decided to euthanize the dog due to financial constraints. This case report describes the atypical morphology and clinical progression of a large canine jejunal leiomyosarcoma, which had similar clinical features as those of human leiomyoma and leiomyosarcoma.

## Introduction

Leiomyosarcomas (LMS) are malignant mesenchymal neoplasms that originate from smooth muscle tissue and, therefore, can arise from any organ containing smooth muscle, such as the gastrointestinal tract, respiratory, urinary tract, genital tract, skin, liver, spleen, and other abdominal organs (1, 2).

Intestinal LMS account for around 30% of canine nonangiogenic, nonlymphogenic intestinal mesenchymal tumors (2–6). Compared to the very low incidence of intestinal LMS in human beings, the incidence rate of canine intestinal LMS is relatively high (2, 6, 7). The jejunum is one of the most commonly affected regions of the gastrointestinal tract affected by LMS (8, 9). The jejunal muscularis propria is usually involved, and the tumors often grow into the bowel lumen, thus inducing obstructive gastrointestinal symptoms, such as the retention of intraluminal gas or fluid (10–13). Several retrospective studies have reported the clinical and morphological characteristics, diagnosis, and prognosis of canine intestinal LMS (14–17). The typical macroscopic morphology of LMS is characterized by large single formation tumors that are larger than 5 cm, with imprecise boundaries that invade the mucosa, muscular layer, and adjacent tissues (16, 17). In human medicine, LMS are usually characterized as a soft, fleshy, ill-defined mass with hemorrhage, necrosis or cystic change (18). With regard to various morphological characteristics that have been previously studied, the size of LMS is considered an important prognostic factor because it has been found that larger tumor size is independently correlated with decreased survival in human beings (19, 20).

This case report describes an atypical morphology and clinical progression of jejunal canine LMS, which was characterized by being large, multiloculated, pseudocystic and fistulated. To the author's knowledge, this is the first case showing an atypical morphology of LMS and its clinical significance.

## Case Description

**Figure 1:**
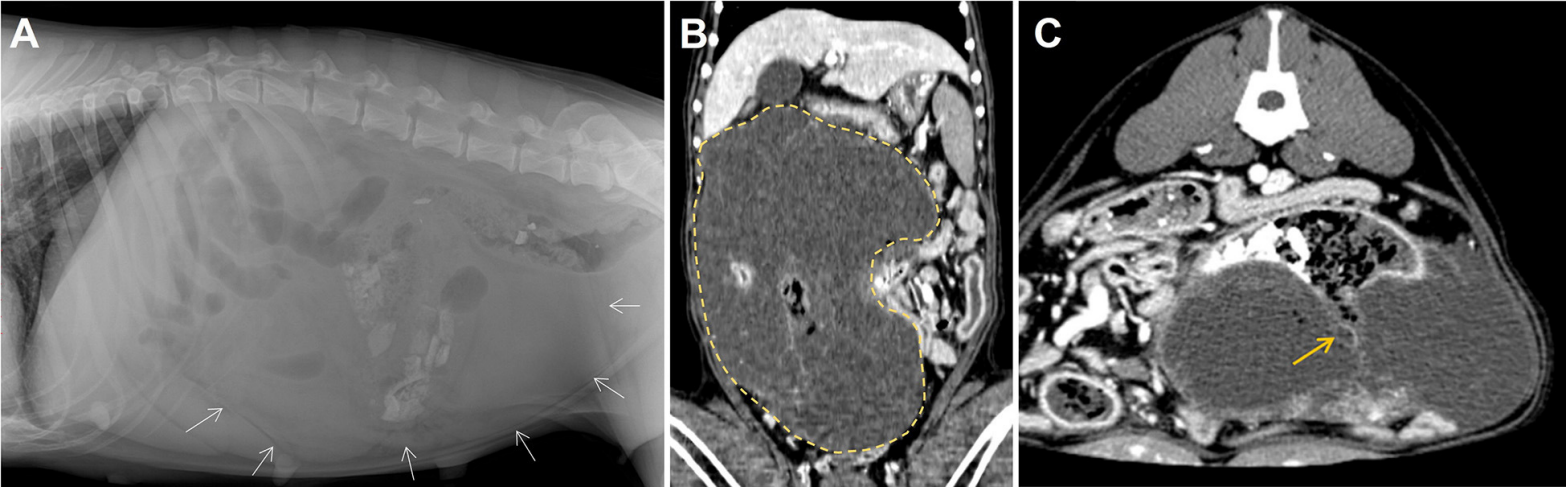
Diagnostic images at presentation. Dorsoventral **(A)** abdominal radiographs revealed a large soft tissue opacity located at the mid abdomen (white arrows). Post-contrast dorsal **(B)** and transvers **(C)** computed tomography scanning images of the mass (yellow dotted line, size of 29 × 19 × 11 cm). Connections between the cavity within the mass and the intestinal lumen represent the presence of fistulation (yellow arrow).

A 10-year-old, 29 kg sexually intact female Rottweiler dog was referred for evaluation of persistent vomiting, anorexia, and lethargy. The referring veterinarian suspected an ovarian tumor based on abdominal ultrasound, which showed a large, cystic, pedunculated intra-abdominal mass. On presentation, the dog was depressed, and had a body condition score of 3/9. Complete blood count and serum biochemistry tests revealed thrombocytopenia (113 K/μL; reference range, 148-484 K/μL) and elevated C-reactive protein levels (210 mg/dL; reference range, 0–35 mg/dL). Plain abdominal radiography (Figure 1A) showed a large mass at the mid-right abdomen, displacing intestinal loops laterally and to the left, and an irregular shape of radiopaque materials. On abdominal ultrasound, an ill-defined, multiloculated cystic mass was identified. Abdominal computed tomography (CT) scan demonstrated a well-demarcated, multiloculated, large mass (29 × 19 × 11 cm) with fluid-filled cavities (Figure 1B). The cavities were separated by thin walls, and numerous various-sized gas opacities were detected within the mass. The jejunum passed through the mass transversely, and a small portion of the jejunum appeared to be fistulated to the cavity within the mass (Figure 1C). Several lymph nodes, including axillary, pancreaticoduodenal, splenic, hepatic, periportal, mesenteric, and colic lymph nodes, were slightly enlarged on CT imaging. Based on CT findings, the mass was suspected to be a small intestinal tumor, and the decision was made to surgically remove the tumor and attach the jejunum via jejunal resection and anastomosis. The owner opted for surgical removal and histopathologic diagnosis of the mass and adjacent enlarged lymph nodes.

**Figure 2:**
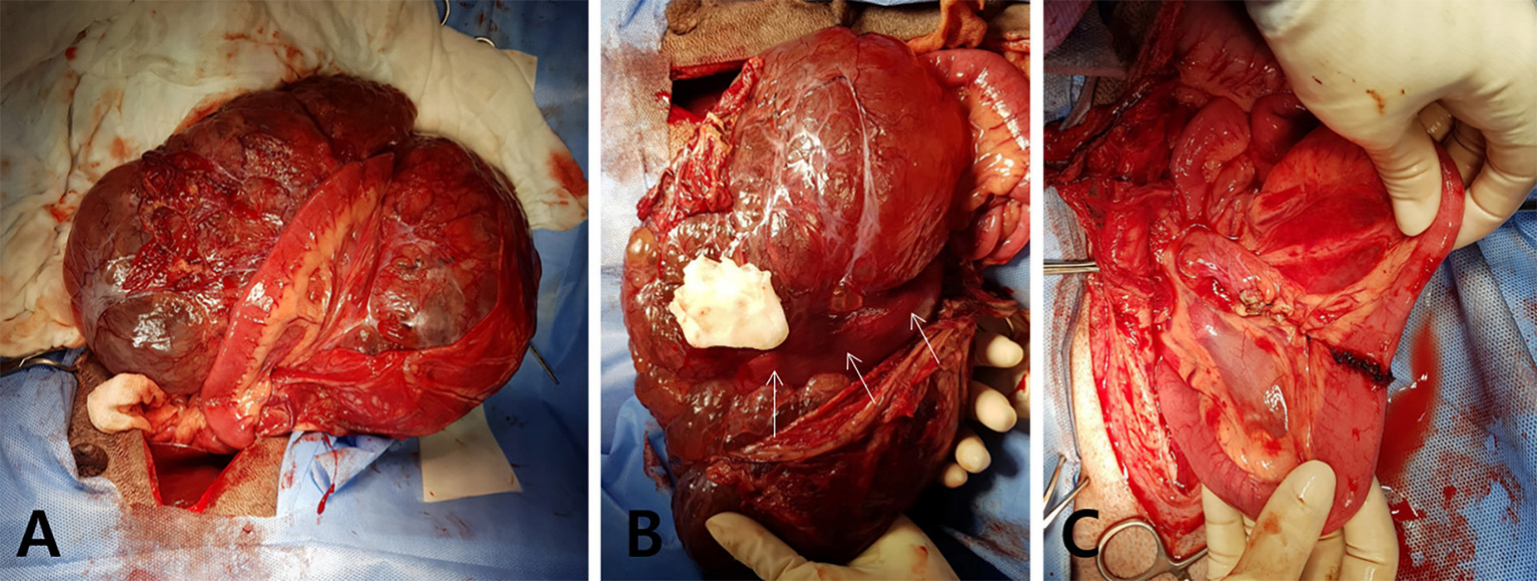
Macroscopic images during surgery. **(A)** Giant intraperitoneal mass is exposed intraoperatively with adherent jejunum. **(B)** The giant, multiloculated cystic mass is located at the jejunal mesentery and attached to the mesenteric border of the surrounding jejunal segment (white arrows). **(C)** The mass and the adhered jejunal segment are completely removed via the jejunal resection and anastomosis.

For the surgical removal, the patient was premedicated with cefazoline (30 mg/kg, intravenous [IV]), famotidine (1 mg/kg, IV), butorphanol (0.2 mg/kg, IV), and midazolam (0.3 mg/kg, IV), followed by anesthesia induction with propofol (6 mg/kg, IV). The patient was intubated, and anesthesia was maintained with isoflurane in oxygen. A ventral midline celiotomy incision was made. During the exploration of the abdominal cavity, a large mass attached to jejunal segments was exteriorized from the abdominal cavity (Figure 2A). The mass appeared multiloculated, cystic, and occupied the jejunal mesenteric region firmly adhering to the associated jejunal segment (Figure 2B). The giant mass was completely removed with the adjacent mesentery and the affected jejunal segments via jejunal resection and end-to-end anastomosis with a 5-cm surgical margin in both oral and aboral directions (Figure 2C). The incisional biopsies were taken from the two enlarged adjacent mesenteric lymph nodes for histopathologic examination. After verifying the absence of leakage from anastomosis site, the abdomen was extensively lavaged with sterile saline and closed routinely. The dog recovered from anesthesia without any complications. Postoperative analgesia was provided by a bolus administration of both fentanyl (0.004 mg/kg, IV) and lidocaine (0.5 mg/kg, IV), followed by a continuous rate infusion of fentanyl (0.004 mg/kg/h, IV) and lidocaine (1.2 mg/kg/h, IV) for 24 h. Postoperatively, the patient was managed with fluid therapy and medications, including tramadol (4 mg/kg, PO) and famotidine (1 mg/kg, PO) twice daily for 7 days. The dog recovered rapidly and was discharged on the third postoperative day without any significant clinical symptoms.

**Figure 3:**
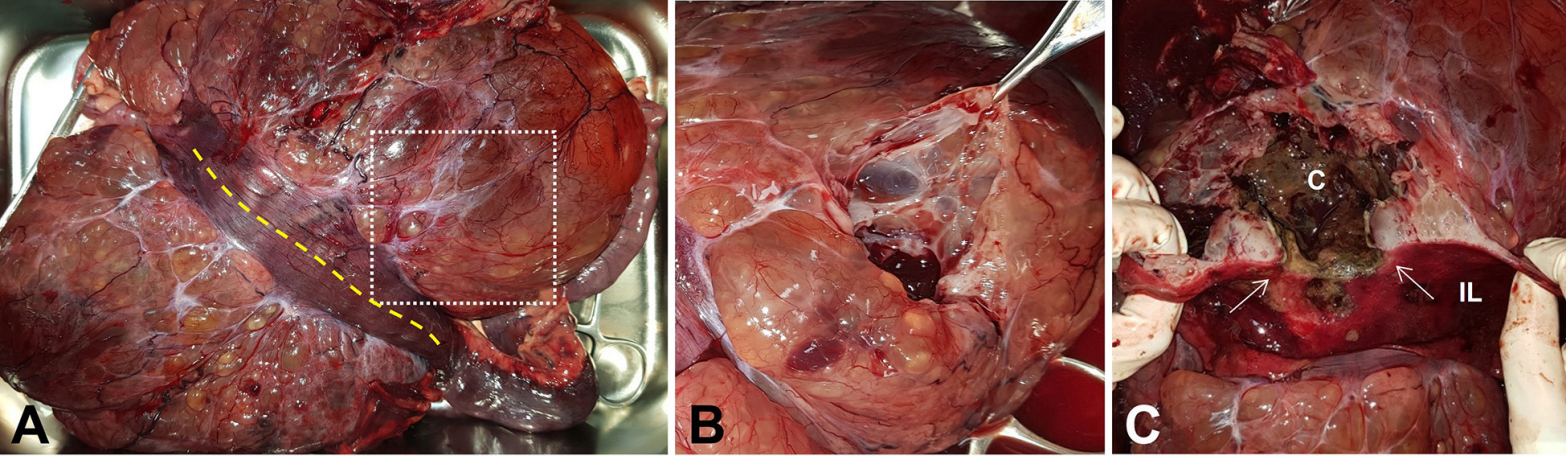
Gross photographs of the resected mass and jejunal segment. **(A)** Antimesenteric side view shows the mass with multicystic appearance. **(B)** The cut surface of the mass indicated as white dotted square in **(A)** reveals polycystic changes. **(C)** Oval-shaped fistula (white arrows) with surrounding mucosal ulceration is exposed through jejunal incision indicated as yellow dotted line in **(A)**. The cut surface of the fistulated intestinal wall reveals the exophytic growth pattern of the mass and the connection between intestinal lumen and necrotic cavity located at the core of the mass. C, cavity; IL, intestinal lumen.

On gross examination, the giant mass was 29 × 19 × 11 cm in size, weighed 2.4 kg, and represented a multiloculated cystic appearance covered with thin fibrous tissue. The mesenteric part of the jejunal segment that enclosed the mass had spread to most of the associated mesentery (Figure 3A). The cut surface of the multiloculated region revealed a polycystic mass that was filled with clear, yellow or serosanguineous fluid (Figure 3B). When the jejunal lumen was exposed by incising the antimesenteric border of the jejunal segment, an oval-shaped ulceration of 11 × 6 mm in size was identified on the jejunal wall. Through the ulcerated area, a fistula was connected to the large cavity, which was filled with dark, red, necrotic contents. The cut surface of the fistulated intestinal wall showed that the mass was growing in an exophytic fashion, expanding outwards from the muscularis layer (Figure 3C). The cystic cavities were located at the core of the mass and were lined by a greenish brown necrotic membrane.

**Figure 4:**
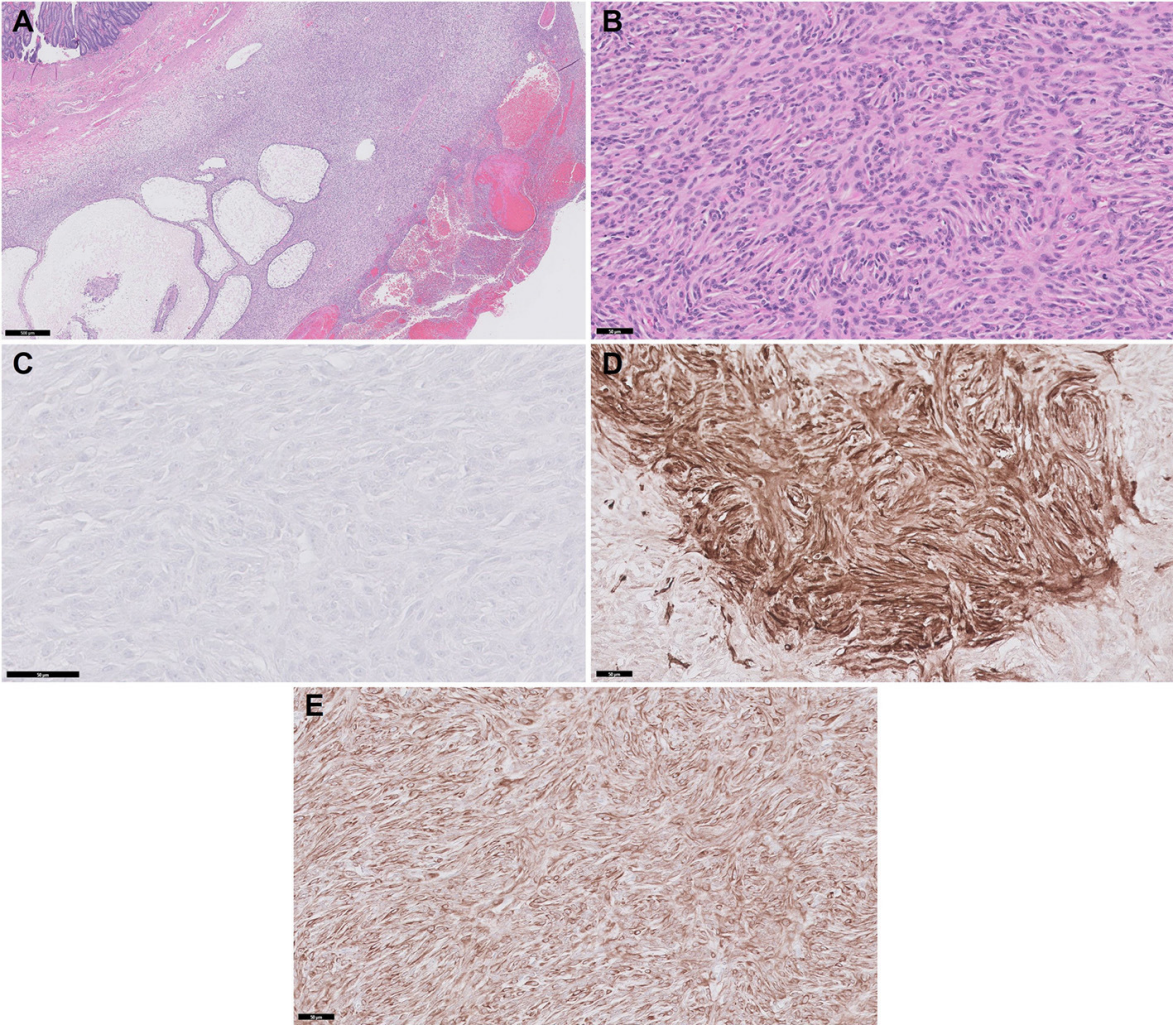
Histopathology and immunohistochemistry (IHC) of the jejunal mass. **(A)** The spindloid neoplastic cells are haphazardly arranged in short bundles and streams with multiple pseudocystic structures. HE stain, Bar, 500 μm. **(B)** The neoplasm is composed of spindle cells with indistinct cell borders, wispy eosinophilic cytoplasm and oval nucleoli. HE stain, Bar, 50 μm. **(C)** Neoplastic cells demonstrate negative labeling with CD117. IHC, DAB chromogen, Bar, 50 μm. **(D)** Neoplastic cells show strong cytoplasmic labeling with Smooth muscle actin. IHC, DAB chromogen, Bar, 50 μm. **(E)** Neoplastic cells show strong cytoplasmic labeling with Desmin. IHC, DAB chromogen, Bar, 50 μm.

The excised mass and affected jejunal segments were examined histologically. An encapsulated, expansile neoplasm was composed of spindle to stellate cells that were haphazardly arranged in short bundles and streams with multiple variably sized fluid-filled pseudocystic structures. The neoplastic cells expanded into the mesentery but did not infiltrate the submucosa and the mucosa (Figure 4A). The resection margins of the tumor, mesentery, and jejunum were free of neoplastic cells. The neoplastic cells had indistinct cell borders, wispy eosinophilic cytoplasm, and oval nucleoli with moderate anisocytosis and anisokaryosis (Figure 4B). The mitotic index was 3 per 10 high-power fields. Immunohistochemistry labeling for CD117 was diffusely negative, which ruled out gastrointestinal stromal tumor (Figure 4C). Neoplastic cells demonstrated strong cytoplasmic labeling for smooth muscle actin (Figure 4D) and desmin (Figure 4E). Based on the gross, histologic, and immunohistochemical results, the jejunal neoplasm was consistent with atypical type of leiomyosarcoma. The histopathological findings of the lymph nodes revealed no evidence of neoplasia but showed reactive hyperplasia. Considering that the tumor was completely removed and there was no evidence of metastasis, adjunctive chemotherapy was not indicated in this dog.

At 6 months post-operatively, the owner reported a recurrence of anorexia in the patient. Abdominal radiology was performed at a veterinary referral hospital, revealing an abdominal mass suspected of being a recurrence of the previous leiomyosarcoma. The owner declined further treatments and opted to euthanize the patient due to financial constraints and concerns for the patient's quality of the life.

## Discussion

The jejunal LMS mass in this dog showed a relatively large size (29 × 19 × 11 cm) compared to the canine intestinal LMS of its type that has been reported thus far. According to previous reports, canine gastrointestinal LMS and leiomyoma (LM) have been reported to be generally larger than 5 cm (16). To our knowledge, up until now, there have been no reports of similar giant-sized intestinal LMS in small animals, even though an 18-cm LMS mass was previously identified in the peritoneum of a dog (21). One reason for such a large tumor in the case reported herein is the exophytic growth of the LMS. Smooth muscle-originating tumors of the gastrointestinal tract frequently extend transmurally thus obstructing the intestinal lumen (13, 22), although, sometimes they bulge out of the serosa in an exophytic fashion (13). These tumors usually then lead to obstructive gastrointestinal symptoms, which could enable the owners to recognize symptoms of the tumors before they grow to an enormous size, except in cases where a tumor occurs in the cecum and is positioned in such a way that it does not limit the passage of intestinal contents (6). In contrast, in the case presented herein, the jejunal LMS expanded outward from the muscular layer as a large extraluminal mass without disrupting the luminal patency; therefore, the tumor did not cause clinical symptoms for a long time. Thus, the owner would not have recognized the symptoms of LMS at an early stage until the giant tumor eventually compressed the adjacent organs and paraneoplastic syndrome had developed, including cancer cachexia and thrombocytopenia. The morphological features of the mass presented herein, which caused the late onset of symptoms and delayed diagnosis, may explain the development of this giant jejunal LMS.

Common morphological characteristics of canine intestinal LMS and LM are that they are typically solid masses and polypoid tissue that often contain hypo/anechoic cavities (16). The formation of hypo/anechoic cavities may be associated with the central degradation of the tumor caused by ischemic central necrosis due to rapid tumor growth outstripping its blood supply (10, 23). Myers et al. reported that 75% of gastrointestinal LMS larger than 3 cm have sonolucent regions, thereby suggesting the presence of central necrosis (12). The dog in the present study also had similar fluid-filled cavities located in the center of the tumor. Considering the giant size of the tumor, ischemic central necrosis was likely going to occur due to the lack of blood supply to the core and due to the formation of a fibrous capsule around the tumor, where the fibrous capsule could reduce the tumor's vascularization and promote ischemic changes (24). Additionally, a fistulous connection between the cavity and intestinal lumen might lead to leakage of the intestinal contents into the cystic cavities, which can induce infection and inflammation and can create central necrosis and intra-cavity exudate production.

The most distinctive feature in the present case is that the multiloculated pseudocystic mass had expanded around the central cavities, unlike the common morphology of canine intestinal LMS. The multiloculated LMS of this dog is the first case reported in small animals that we are aware of, despite being commonly reported in LMS and LM of the uteri of human beings (25, 26). A fair number of the previously reported human uterine LMS exhibit multilocular pseudocystic morphology, which often makes them confused with ovarian tumors (26). Hydropic degeneration, which is characterized by the intra-tumoral accumulation of edematous fluid, has been thought to be the main cause of inducing the pseudocystic morphology of human uterine LM; however, the etiology of the degeneration remains unclear (26, 27). Rarely, the pseudocystic morphology may be associated with myxoid degeneration that produces mucinous materials, but most LMS are represented by hydropic degeneration in which clear, yellow, and hemorrhagic fluid accumulated in the multiloculated pseudocystic spaces (26). In the case presented herein, hydropic degeneration was identified as multiple variably sized fluid-filled spaces within the tumor, and the fluid in these spaces was clear, yellow or serosanguineous. These features make the intestinal LMS in the present case unique, as it represents common characteristics of canine intestinal LMS, such as forming hypo/anechoic cavities, but does not have the morphological features of human uterine LMS/LM, such as multiloculation occurring by hydropic degeneration. In addition, this multiloculated encapsulated mass surrounding the cavities may have prevented septic peritonitis from intestinal perforation and leakage of intestinal contents. Intestinal perforation and subsequent leakage of intestinal contents have been previously described in dogs with intestinal LMS (14, 15). This perforation and leakage of intestinal contents can immediately lead to peritonitis and subsequent sepsis and can be present in 50% of dogs with intestinal LMS (14). However, in the present case, despite a sizable intestinal ulcer-related perforation, the leaked intestinal contents were accumulating only in the cavity at the core of the mass without leaking into abdominal cavity. This morphological feature enabled prolonged intestinal perforation and leakage without obvious clinical symptoms and thus formed a fistula connecting the LMS and the intestinal lumen.

The prognostic factors of canine gastrointestinal LMS have not been characterized because the presence of metastases and histologic features have not clearly impacted prognosis (6, 8, 14). In human studies, the giant size of an LMS has clinical significance because the size of the tumor has been reported as the only reliable prognostic indicator of LMS in human beings (19, 20). Miyajima et al. reported that LMS size is correlated independently with decreased survival in a multivariate analysis of 267 human patients (20). In another human study reporting small bowel LMS, a similar trend was reported because the average tumor size in the surviving group was 9.3 cm, compared to an average tumor size of 13.8 cm in the group who died within 5 years; thus, larger tumor size may be a negative prognostic indicator (19). In veterinary medicine, to our knowledge, there are currently no studies demonstrating that prognosis is related to the size of intestinal LMS; however, dogs that have an early diagnosis of gastrointestinal smooth muscle tumor(s) have been reported to show higher survival rates (28). The dog in the present study was euthanized 6 months after surgery due to high suspicion for LMS recurrence. The survival of the case represented herein is shorter than that previously reported because previous reports have shown a postoperative median survival time ranging from 10 to 21.3 months for intestinal LMS (8, 14). Canine intestinal LMS are not regarded as being highly aggressive since the metastasis and mesenteric invasion observed during diagnosis or surgery may not negatively affect the prognosis (14, 29). The giant size of LMS, as seen in this case, may be associated with a worse prognosis and may include a high recurrence rate and short post-surgical survival time; therefore, the size of LMS tumors in dogs should be considered as a potential negative prognostic indicator, similar to human studies.

## Conclusion

The canine jejunal LMS in the present report had atypical macroscopic and morphological features, including the giant size, multiloculated and pseudocystic and exhibiting intestinal perforation without peritonitis and exophytic growth without interfering the luminal patency. The morphological features of the case presented herein, in contrast to previously reported features in dogs with intestinal LMS, are the first reported morphological characteristics of canine LMS that have similar features to LM and LMS in human beings. Furthermore, especially in the case of giant canine intestinal leiomyosarcoma, the possibility of unfavorable prognosis with regard to size of the tumor should be considered.

## Data Availability Statement

The original contributions presented in the study are included in the article/supplementary material, further inquiries can be directed to the corresponding author/s.

## Author Contributions

M-YK did the clinical examination and assisted the surgery and followed up the patient after surgery. H-JH was the main surgeon of this study. JL and KM did pathological analysis. M-YK, JL, KM, and H-JH analyzed the case and drafted the manuscript. All authors have read and approved the final version of the manuscript.

## Funding

This work was supported by Konkuk University in 2021.

## Conflict of Interest

Authors JL and KM were employed by Idexx Laboratories. The remaining authors declare that the research was conducted in the absence of any commercial or financial relationships that could be construed as a potential conflict of interest.

## Publisher's Note

All claims expressed in this article are solely those of the authors and do not necessarily represent those of their affiliated organizations, or those of the publisher, the editors and the reviewers. Any product that may be evaluated in this article, or claim that may be made by its manufacturer, is not guaranteed or endorsed by the publisher.

## References

[1] Cooper BJ Valentine BA. Tumors of muscle. In: Meuten DJ editor. Tumors in Domestic Animals, 5th ed. Ames: Wiley Blackwell (2017). p. 425–66. 10.1002/9781119181200.ch11

[2] Hayes S Yuzbasiyan-Gurkan V Gregory-Bryson E Kiupel M. Classification of canine nonangiogenic, nonlymphogenic, gastrointestinal sarcomas based on microscopic, immunohistochemical, and molecular characteristics. Vet Pathol. (2013) 50:779–88. 10.1177/0300985813478211 23456969

[3] Bruecker KA Withrow SJ. Intestinal leiomyosarcoma in six dogs. J Am Anim Hosp Assoc. (1988) 24:281–4.

[4] Chen HC Parris LS Parris RG. Duodenal leiomyosarcoma with multiple metastases in a dog. J Am Vet Med Assoc. (1984) 184:1506. 6735875

[5] Cohen M Post GS. Nephrogenic diabetes insipidus in a dog with intestinal leiomyosarcoma. J Am Vet Med A. (1999) 215:1818–20. 10613214

[6] Maas CPHJ Haar GT Gaag IVD Kirpensteijn J. Reclassification of small intestinal and cecal smooth muscle tumors in 72 dogs: clinical, histologic, and immunohistochemical evaluation. Vet Sur. (2007) 36:302–13. 10.1111/j.1532-950X.2007.00271.x 17547593

[7] Singh Z. Leiomyosarcoma: A rare soft tissue cancer arising from multiple organs. J Cancer Res Pract. (2018) 5:1–8. 10.1016/j.jcrpr.2017.10.0029211528

[8] Kapatkin AS Mullen HS Matthiesen DT Patnaik AK. Leiomyosarcoma in dogs: 44 cases (1983-1988). J Am Vet Med Assoc. (1992) 201:1077–9. 1429139

[9] Patnaik AK Hurvitz AI Johnson GF. Nonlymphoid intestinal neoplasia in 32 dogs and 14 cats. Vet Pathol. (1977) 14:547–55. 10.1177/030098587701400602 579266

[10] Chen JJ Changchien CS Chiov SS. Various sonographic patterns of smooth muscle tumors of the gastrointestinal tract: a comparison with computed tomography. J Ultrasound Med. (1992) 11:527–31. 10.7863/jum.1992.11.10.527 1404582

[11] Frost D Lasota J Miettinen M. Gastrointestinal stromal tumors and leiomyosarcomas in the dog: a histopathologic, immunonhistochemical, and molecular genetic study of 50 cases. Vet Pathol. (2003) 40:42–54. 10.1354/vp.40-1-42 12627712

[12] Myers NC Penninck DG. Ultrasonographic diagnosis of gastrointestinal smooth muscle tumors in the dog. Vet Radiol Ultrasound. (1994) 35:391–7. 10.1111/j.1740-8261.1994.tb02059.x

[13] Subramanyam BR Balthazar EJ Raghavendra BN Madamba MR. Sonography of exophytic gastrointestinal leiomyosarcoma. Gastrointest Radiol. (1982) 7:47–51. 10.1007/BF01887605 7060873

[14] Cohen M Post GS Wright JC. Gastrointestinal leiomyosarcoma in 14 dogs. J Vet Intern Med. (2003) 17:107–10. 10.1111/j.1939-1676.2003.tb01331.x 12564735

[15] Fragelecova L Skoric M Fictum P Husnik R. Canine gastrointestinal tract tumors: a retrospective study of 74 cases. Acta Bet Brno. (2013) 82:387–92. 10.2754/avb20138204038725181273

[16] Hobbs J Suther-Land J Penninck D Jenning S Barber L Barton B. Ultrasonographic features of canine gastrointestinal stromal tumors compared to other gastrointestinal spindle cell tumors. Vet Radiol Ultrasound. (2015) 56:432–8. 10.1111/vru.12253 25846814

[17] Leandro RM Freitas FP Sa LRM. The importance of clinical, histopathological and immunohistochemical marking for differential diagnosis of non-hematopoietic gastrointestinal mesenchymal neoplasm in dog: literature review. Braz J Vet Res Anim Sci. (2017) 54:287–97. 10.11606/issn.1678-4456.bjvras.2017.128846

[18] Marko J Wolfman DJ. Retroperitoneal Leiomyosarcoma from the radiologic pathology archives. RadioGraphics. (2018) 38:1403–20. 10.1148/rg.2018180006 30207936 PMC6166742

[19] Aggarwal G Sharma S Zheng M Reid MD Crosby JH Chamberlain SM. Primary leiomyosarcomas of the gastrointestinal tract in the post-gastrointestinal stromal tumor era. Ann Diagn Pathol. (2012) 16:532–40. 10.1016/j.anndiagpath.2012.07.005 22917807

[20] Miyajima K Oda Y Oshiro Y Tamiya S Kinukawa N Masuda K. Clinicopathological prognostic factors in soft tissue leiomyosarcoma: a multivariate analysis. Histopathology. (2002) 40:353–9. 10.1046/j.1365-2559.2002.01361.x 11943020

[21] Ochoa-Amaya J Zambrano D Roque-Rodriguez A Queiroz-Hazarbassanox N Dagli MZ. Peritoneal leiomyosarcoma in a canine: case report. Rev MVZ Cordoba. (2019) 24:7378–83. 10.21897/rmvz.1363

[22] Hurov L. Perforating ulcers of the small intestine lymphosarcoma and fibrosarcoma complications. Can Vet J. (1962) 3:385–7. 17421556 PMC1585970

[23] Rowley VA Cooperberg PL. The ultrasonographic appearance of abdominal leiomyosarcoma. J Can Assoc of Rad. (1982) 33:94–7. 7107682

[24] Iacono S Serio G Fugazzola C Zamboni G Bergamo Andreis IA Jannucci A. Cystic islet cell tumors of the pancreas. A clinico-pathological report of two nonfunctioning cases and review of the literature. Int J Pancreatol. (1992) 11:199–208. 10.1007/BF02924187 1325529

[25] Kadkhodayan S Yousefi Z Hasanzadeh M Sistani NS. Homaee F. Leiomyosarcoma with Unusual Macroscopic Features: A Case Report. (2015). p. 433–6, vol. 3.

[26] Yorita K Tanaka Y Hirano K Kai Y Arii K Nakatani K. subserosal, pedunculated, multilocular uterine leiomyoma with ovarian tumor-like morphology and histological architecture of adenomatoid tumors: a case report and review of the literature. J Med Case Rep. (2016) 10:352. 10.1186/s13256-016-1167-1 27998309 PMC5175316

[27] Coard K Plummer J. Massive multilocular cystic leiomyoma of the uterus: an extrem example of hydropic degeneration. South Med J. (2007) 100:309–12. 10.1097/01.smj.0000257639.52322.7d 17396738

[28] Crawshaw J Berg J Sardinas JC Engler SJ Rand WM Ogilvie GK. Prognosis for dogs with nonlymphomatous, small intestinal tumors treated by surgical excision. J Am Ani Hosp Assoc. (1998) 34:451–6. 10.5326/15473317-34-6-451 9826278

[29] Spyckerelle C. Leiomyosarcoma canin: généralités, diagnostic, traitement et pronostic. Le Point vétérinaire. (2011) 42:54–5.

